# Leadership and Human–AI Collaboration: A Measurement Scale

**DOI:** 10.3390/bs16071208

**Published:** 2026-07-17

**Authors:** Julio César Acosta-Prado, José Pablo Camargo, Rodrigo Arturo Zárate-Torres, C. Fabiola Rey-Sarmiento

**Affiliations:** 1 Departamento de Ingeniería, Pontificia Universidad Católica del Perú, Lima 15088, Peru; 2 Departamento de Investigación, Colegio de Estudios Superiores de Administración—CESA, Bogotá 110311, Colombia; jose.camargo@cesa.edu.co (J.P.C.); rodrigo.zarate@cesa.edu.co (R.A.Z.-T.); fabiola.rey@cesa.edu.co (C.F.R.-S.)

**Keywords:** leadership, human–AI collaboration, measurement scale, psychometric properties

## Abstract

This paper aims to develop and provide the validity and reliability of a measurement scale that establishes the relationship between Leadership and Human–AI Collaboration. An instrumental study was conducted to inform the design and revision of a scale’s psychometric properties. The techniques used for statistical analysis were carried out in three sequences, namely, the first consisted of collecting validity evidence based on test content; the second consisted of an exploratory and confirmatory factor analysis to gather validity evidence based on internal structure; and the third consisted of estimating the test reliability of internal consistency through the omega coefficient. The results show that the proposed measurement scale meets the psychometric properties required of a social-science instrument.

## 1. Introduction

In recent years, AI has established itself as one of the most influential technologies in organizational transformation. It has moved beyond its initial function as an automation tool, progressively integrating into the structure and administrative processes of organizations, data-driven decision-making, and value creation.

Recent literature agrees that the impact of AI cannot be understood solely from a technological perspective, but rather as a sociotechnical phenomenon that reconfigures the relationship between people, systems, and organizational structures (Raisch & Krakowski, 2021; Shrestha et al., 2019). In this sense, AI not only increases efficiency and optimizes operations but also redefines the nature of work, organizational structure, and coordination and communication mechanisms.

However, the adoption and effective use of AI present significant challenges, regardless of the size and sector of the organizations, as they face limitations to its adoption, associated with digitalization gaps, heterogeneous technological capabilities, geographical dispersion, internet access in remote areas, data quality problems, and regulatory frameworks still in consolidation (Sun et al., 2025).

These conditions imply that the implementation of AI is not a linear or homogeneous process but a complex one, dependent on organizational factors that interact and feed back on one another, such as leadership, innovation culture, and continuous learning (Q. Meng et al., 2025).

In the recent literature, the relationship between leadership and collaboration between humans and AI has been addressed using various measurement scales that capture dimensions such as trust in AI systems, the quality of human–machine interaction, and the role of leadership in sociotechnical environments. Instruments such as the Automation Trust Scale proposed by Hoff and Bashir (2015) allow for the assessment of individuals’ willingness to rely on intelligent systems, while approaches such as those of Seeber et al. (2020) conceptualize and operationalize human–AI collaboration in terms of co-creation and joint performance or ethical integration. Also, Larasati et al. (2023) developed and validated an instrument to measure trust attitudes towards AI systems from a layperson (non-expert) perspective.

In parallel, classic leadership scales such as the Multifactor Leadership Questionnaire (MLQ) by Bass and Avolio (1995) have been widely used to analyze how transformational and transactional leadership styles influence the adoption and effective use of intelligent technologies. Likewise, emerging scales associated with digital leadership or e-leadership (Avolio et al., 2020) incorporate dimensions such as technological facilitation, change management, and the promotion of augmented intelligence, positioning the leader as a key player in articulating collaboration between humans and AI (Guillén-Gámez et al., 2026; Perkins et al., 2024). However, no previous study has created a scale that measures human–AI leadership and collaboration, integrated into an explanatory model.

Therefore, this study makes a unique and novel contribution by explicitly connecting two conceptual frameworks that have evolved in parallel—leadership and human–AI collaboration—overcoming the existing fragmentation between scales focused on AI trust/use and those focused on leadership styles. Unlike previous instruments that measure partial constructs (e.g., trust in AI or transformational leadership), this study proposes an original scale specifically designed to capture the intersection between leadership and human-AI collaboration.

The proposed measurement scale redefines leadership not only as a set of general styles or behaviors, but as practices related to the strategy, ethical, sustainable, and organizational management of AI in collaborative contexts, rather than leadership as a general organizational capability.

Methodologically, this study builds upon the explanatory model proposed by Zárate-Torres et al. (2025), which seeks to simultaneously examine how leadership influences the quality and effectiveness of human–AI collaboration, moving beyond descriptive or unidimensional approaches.

In short, this study fills a gap in the literature by providing a validated tool that can be used in future empirical studies, facilitating comparability and knowledge accumulation in leadership applied to human–AI collaborative organizational environments.

Based on the above, this study seeks to develop and provide validity evidence for a measurement scale, based on the explanatory model proposed by Zárate-Torres et al. (2025), which measures the constructs: leadership and human–AI collaboration.

## 2. Literature Review

The explanatory model proposed by Zárate-Torres et al. (2025) measures two constructs. The first identifies Leadership as a relational and dynamic process where the leader acts as a “sense-maker,” facilitating collective understanding of change, managing emerging emotions, and constructing narratives that legitimize the responsible use of new technologies (Goleman, 1995; Weick, 1995; Jarrahi, 2018), articulating the technologic adoption processes (Gignac & Szodorai, 2024; Russell & Norvig, 2010) and conceiving, adopting, and institutionalizing intelligent technologies that profoundly condition collaboration, supervision, and delegation between humans and machines (Adeniyi et al., 2024; Floridi, 2019).

The second Human–AI Collaboration construct is conceptualized as a sociotechnical interaction system in which both agents integrate complementary capabilities to solve complex tasks. Within this framework, humans contribute contextual judgment, interpretation, and meaning-making (Elfighi, 2025; L. Meng et al., 2024; Oesch, 2024; Pan et al., 2024), while artificial intelligence contributes processing speed, analytical capacity, and the generation of alternatives (Jiang et al., 2022; Kaplan & Haenlein, 2019; EC HLEG AI-European Commission, 2019), thus establishing a mutually supportive, performance-oriented relationship (Gignac & Szodorai, 2024). However, the effectiveness of this collaboration depends on the nature of the task and the degree of functional complementarity between the two agents (Florea & Croitoru, 2025; Vaccaro et al., 2024; Bredeweg & Kragten, 2022).

Based on these considerations, a set of relationships is established that, covered by the proposed definition of the Human–AI Collaboration and the proposed explanatory model, determines both the catalytic role of the Leadership and their influence on the use of emerging technologies in organizations (Alshaibani et al., 2025; Baez, 2025; Bevilacqua et al., 2025; Bock & von der Oelsnitz, 2025; Vargas Portillo, 2025; Hossain et al., 2025).

The explanatory model seeks to elucidate the conditions and consequences underlying Human–AI Collaboration in the organizational environment, considering the influence of Leadership and the possible results. The model is framed within the socio-technical approach and in the so-called hybrid models. This model analyses the complementary nature, and in no case discordant, of the intelligences, such as human and artificial, in the formation and adaptation of organizations under conditions of uncertainty and rapid change. Therefore, the objective is to reflect the way in which leadership acts as the axis that brings together human and technological systems to work together (Zárate-Torres et al., 2025).

### 2.1. Leadership

In this study, Leadership refers specifically to leadership practices related to the strategy, ethics, sustainability, and organizational management of AI in collaborative contexts, rather than to leadership as a general organizational capability. Leadership in the digital age constitutes an emerging form of adaptive leadership that seeks to maximize the value of the complementarity between human and technological capabilities, while preserving human oversight of decision-making and the ethical orientation of the organization (López-Figueroa et al., 2025; Hossain et al., 2025; Dellermann et al., 2019). The advancement of intelligent technologies demands that leaders and organizations develop unprecedented levels of adaptability and agility to understand and manage technological innovations and the evolving market dynamics, through the strategic management of technology, sustainability, and the organization’s environmental responsibility (Bock & von der Oelsnitz, 2025; Florea & Croitoru, 2025; Bevilacqua et al., 2025).

#### 2.1.1. Leadership and Strategic Management (LSM)

Leadership in AI requires articulating a strategic vision that achieves structural coupling between technology and systemic objectives. The absence of this articulation leads to fragmented initiatives and high organizational resistance (Krishnan, 2025). Aligning leadership with business strategy demands skills to manage complexity and promote transdisciplinary collaboration, breaking down traditional administrative silos. Leadership assumes the critical function of reducing uncertainty; this implies developing the capacity to communicate with and support employees during the transition, creating environments of psychological safety (Edmondson, 1999), enabling the organizational system to absorb technological disruption without fracturing its social fabric (Ji et al., 2026).

#### 2.1.2. Sustainable Leadership and Environmental Responsibility (SLER)

The relationship between AI and sustainability is examined from an ambivalent perspective. On the one hand, AI offers disruptive opportunities to optimize processes and improve resource efficiency, directly contributing to the achievement of environmental and social sustainable development goals (Vinuesa et al., 2020; Chowdhury et al., 2022). On the other hand, AI implementation can accelerate system entropy through increased energy consumption, opacity in decision-making, and the exacerbation of preexisting inequalities (Falk & van Wynsberghe, 2024). This duality implies that sustainability should not be conceived as an automatic byproduct of technology, but rather as an exercise in responsibility (Wu & Zhang, 2024), ensuring that technology brings order and balance to the business ecosystem (Schwaeke et al., 2025).

### 2.2. Human–AI Collaboration

The collaboration between humans and AI is defined as a complementary interaction process in which human cognitive abilities—such as critical judgment, creativity, and ethical decision-making—are integrated with the analytical and data-processing capabilities of AI systems to improve performance, problem-solving, and decision-making in diverse organizational and social contexts (Li et al., 2023; Bankins et al., 2024; Chowdhury et al., 2022). From this perspective, AI does not replace human intelligence but rather acts as a support resource that amplifies human skills and fosters more efficient and accurate work processes (Liu & Shen, 2025; Tang et al., 2022; Haesevoets et al., 2021).

#### 2.2.1. Nature of the Interaction (NI)

The relationship between humans and intelligent systems must go beyond the idea of replacement and be understood from a perspective of systemic complementarity (Raisch & Krakowski, 2021; Shrestha et al., 2019). This perspective recognizes the advantages of AI in structured and data-intensive tasks. However, humans retain distinct capacities for contextual judgment and the interpretation of ambiguous situations—essential faculties for decision-making in uncertain environments (Korteling et al., 2021; Tambe et al., 2019). This distinction is not merely theoretical; it constitutes a reconfiguration of the internal organizational structure, where the biological and the algorithmic interact and reinforce each other (Chen et al., 2024).

#### 2.2.2. Productivity and Efficiency (PE)

The notion of productivity in the AI era must be rescued from the reductionist view of output per unit of time. From a complexity perspective, Okafor et al. (2023) suggest that efficiency is not just a financial indicator, but rather the organization’s capacity to generate resonance (Rosa, 2019) with its environment. AI does not simply accelerate processes; it alters the ontology of value creation, facilitating the transition of companies from rigid structures to real-time response models that can learn from their own disturbances and reduce organizational entropy (Raisch & Krakowski, 2021; Georgescu-Roegen, 1971).

#### 2.2.3. User Experience and Acceptance (UXA)

The acceptance of AI has become a key factor in the success of its organizational implementation, going beyond mere technical installation. Technological adoption is closely linked to the perceived usefulness of users and the degree of integration of the tool into daily practices (Davenport & Ronanki, 2018; Dwivedi et al., 2021); this process is understood as structural coupling (Han & Ko, 2025), where AI only generates real value when it effectively aligns with the stakeholders who adopt it, transforming the internal communications network (Wang & Yu, 2026; Rawas, 2024).

#### 2.2.4. Impact on the Role of the Employee (IRE)

AI transforms the role of the worker by radically altering the nature of tasks and the skills required for organizational survival. This process should not be understood as a linear replacement of human employment, but rather as a systemic redistribution of work (Acemoglu & Restrepo, 2019; Frey & Osborne, 2017). This shift implies a redefinition of roles toward functions with analytical, interpretive, and supervisory content. Therefore, routine tasks tend to be automated under the logic of technical efficiency; human functions are oriented toward managing emergent events (Daugherty & Wilson, 2024), activities that require critical judgment, contextual understanding, and decision-making in highly uncertain environments (Chu & Chu, 2026).

#### 2.2.5. Governance and Ethics (GE)

AI governance constitutes a structural dimension of its organizational implementation that transcends technical management to enter the realm of organizational bioethics. As algorithmic systems gain autonomy in critical processes such as personnel selection, resource allocation, and performance evaluation, challenges of opacity and traceability emerge, demanding a return to the foundations of principlism (Beauchamp & Childress, 2019). Thus, AI ethics is not a formal addendum, but an organizational emergency (Mäntymäki et al., 2022).

#### 2.2.6. Innovation and Organizational Transformation (INOT)

AI is consolidating its position as a fundamental driver of organizational innovation, enabling the profound reconfiguration of processes, business models, and decision-making structures (Labib, 2024; Bankins et al., 2024). This transformation does not emerge automatically after technological adoption; innovation is an emergent event that depends on the organizational system’s capacity to absorb, adapt, and scale the use of AI according to strategic objectives. This implies integrating, building, and reconfiguring resources in rapidly changing environments (Bankins et al., 2024; Kolbjørnsrud, 2024). Thus, AI acts as a catalyst, intensifying the demands for organizational learning and continuous experimentation (Cyfert et al., 2025).

In summary, the eight dimensions of analysis were selected and grouped in the literature review of the explanatory model proposed by Zárate-Torres et al. (2025), which establishes that the relationship between leadership and human-AI collaboration requires modeling leadership as a multidimensional factor that shapes the integration, experience, and governance of AI within organizations. The dimensions of Leadership and Strategic Management and Sustainable Leadership and Environmental Responsibility reflect how leaders define the vision, allocate resources, and incorporate ethical and sustainability principles into AI deployment.

These leadership-oriented dimensions can be analyzed as antecedents that influence the outcomes of human-AI collaboration. In turn, dimensions such as Nature of Interaction, Productivity and Efficiency, and User Experience and Acceptance reflect the quality and effectiveness of human–AI collaboration at the operational level. Leadership dimensions seek to predict interaction quality, perceived usefulness, and efficiency improvements, while influencing user acceptance and trust in AI systems.

At a more interpretive level, the dimensions of Employee Role Impact, Governance and Ethics, and Organizational Innovation and Transformation allow us to assess the broader organizational consequences of this relationship. Leadership practices influence how employees redefine their roles in AI-driven environments, whether governance mechanisms ensure responsible and transparent use of AI, and the extent to which human-AI collaboration fosters innovation and organizational transformation.

Taken together, these dimensions can encompass both behavioral and systemic effects. This not only provides a comprehensive framework for examining the relationship between leadership and human–AI collaboration but also for understanding how and through what mechanisms this interconnectedness manifests itself.

## 3. Materials and Methods

An instrumental study was conducted to design and review the psychometric properties of the measurement scale (Montero & León, 2007) that assesses the influence of leadership on human-AI collaboration, based on the explanatory model proposed by Zárate-Torres et al. (2025).

The sample comprised 170 responses obtained from Colombian companies. Organizations of different sizes were represented, ranging from microenterprises with fewer than 10 employees to large firms with more than 200 employees. The participating companies operated across a wide range of sectors in the national economy, including agribusiness, manufacturing, services and consulting, construction, education, the cultural sector, technology, hospitality, transportation and logistics, retail, finance and insurance, healthcare, and mining and energy. The data were collected electronically through a self-administered online survey. All indicators were measured using a positively worded Likert-type scale.

The instrument was composed of nine sections. The first section gathered general information about the company and the respondent responsible for completing the survey. The remaining sections corresponded to the substantive domains of the scale: Nature of the Interaction [NI_1–4], Productivity and Efficiency [PE_1–4], User Experience and Acceptance [UXA_1–4], Impact on the Role of the Employee [IRE_1–3], Governance and Ethics [GE_1–5], and Innovation and Organizational Transformation [INOT_1–4], Leadership and Strategic Management [LSM_1–3], and Sustainable Leadership and Environmental Responsibility [SLER_1–3]. In total, the instrument comprised 30 measurement indicators rated on a five-point scale, where 1 corresponded to “Never,” 2 to “Rarely,” 3 to “Sometimes,” 4 to “Often,” and 5 to “Always.” Appendix A shows the 30 items that make up the measurement scale (Table A1).

All statistical analyses were conducted using the software R Core Team (2026), version 4.6.0. These packages were used to support the estimation of the models, the assessment of reliability, and the examination of the structure of the instrument factors.

The study was conducted in two stages. First, a descriptive analysis of the items was performed to examine response distributions, frequencies, and measures of central tendency and dispersion across the indicators. This stage provided an initial overview of the behavior of the items and allowed the identification of general response patterns within each section of the instrument. Second, the internal structure of the scale was examined through two theoretically defined measurement blocks rather than through a single simultaneous model including all eight substantive dimensions.

The first block was conceptualized as Leadership, which grouped the dimensions associated with strategic leadership and sustainability in the organizational use of artificial intelligence: Leadership and Strategic Management (LSM), and Sustainable Leadership and Environmental Responsibility (SLER). This block captures the extent to which leadership practices guide the strategic adoption of AI, align AI use with organizational goals, and incorporate environmental and sustainability-oriented considerations into AI-related decision-making.

The second block was conceptualized as Human–AI Collaboration, which grouped the dimensions associated with the interaction between human actors and AI systems in organizational settings: Nature of the Interaction (NI), Productivity and Efficiency (PE), User Experience and Acceptance (UXA), Impact on the Role of the Employee (IRE), Governance and Ethics (GE), and Innovation and Organizational Transformation (INOT). This block captures the extent to which AI is integrated into work processes, decision support, user experience, role transformation, ethical governance, and organizational innovation.

Before conducting the exploratory factor analysis (EFA), the adequacy of the correlation matrices was evaluated using the Kaiser–Meyer–Olkin (KMO) index (Kaiser, 1974) and Bartlett’s test of sphericity (Bartlett, 1950). In all EFA estimations, Unweighted Least Squares was used as an extraction method. To provide empirical guidance regarding the number of factors to retain, the Kaiser–Guttman criterion, the Scree test (Cattell, 1966), and parallel analysis (Horn, 1965) were examined. However, these procedures were treated as heuristic criteria rather than strict decision rules, and the retained exploratory solution was selected based on statistical performance, substantive interpretability, and theoretical coherence. To facilitate interpretation of the factor structure, an oblique Oblimin rotation was applied.

The confirmatory factor analyses (CFA) were performed in both measurement blocks using the weighted least squares mean and variance adjusted (WLSMV) estimator, with robust standard errors and a scaled-shifted test statistic, given its suitability for ordinal indicators. Model fit was assessed using the Comparative Fit Index (CFI), with values above 0.90 considered acceptable (Keith, 2019); the Tucker–Lewis Index (TLI), with values above 0.90 indicating adequate fit (Keith, 2019; Schumacker & Lomax, 2016); the Root Mean Square Error of Approximation (RMSEA), with values below 0.08 considered acceptable (Keith, 2019; Schumacker & Lomax, 2016); and the Standardized Root Mean Square Residual (SRMR), for which values below 0.08 indicate adequate fit (Hu & Bentler, 1999; Keith, 2019).

The EFA and CFA were conducted using the same sample of 170 responses. However, the CFA model comprising six dimensions of Human–AI Collaboration was not derived from the exploratory results; it was specified a priori based on the theoretical structure proposed by Zárate-Torres et al. (2025). The CFA was therefore used to evaluate the empirical plausibility of the pre-established six-factor measurement model and to compare it with alternative specifications, including the reduced structure derived from the EFA. Because no independent validation sample was available, these results represent within-sample evidence rather than an independent cross-validation of the measurement structure.

Discriminant validity was assessed using the Fornell–Larcker criterion and the heterotrait–monotrait ratio of correlations (HTMT). According to the Fornell–Larcker criterion, discriminant validity was considered supported when the square root of the average variance extracted (AVE) for each factor exceeded its absolute correlation with the remaining factors (Fornell & Larcker, 1981). For HTMT, values below 0.85 were interpreted as satisfying the strict criterion for discriminant validity, values between 0.85 and 0.90 as satisfying the more flexible criterion, and values above 0.90 as indicating insufficient discriminant validity between the corresponding factors (Henseler et al., 2015).

Internal consistency reliability was assessed using Cronbach’s alpha, ordinal alpha, and omega coefficients. Because omega coefficients are especially appropriate in the context of confirmatory factor analysis, as they account for standardized factor loadings and measurement error, they were considered the primary indicators of internal consistency. Values equal to or greater than 0.70 for each dimension were interpreted as evidence of satisfactory reliability (Nunnally & Bernstein, 1994).

To examine the bivariate associations among all dimensions included in the instrument, composite scores were calculated as the mean of the items corresponding to each of the eight dimensions: Leadership and Strategic Management, Sustainable Leadership and Environmental Responsibility, Nature of the Interaction, Productivity and Efficiency, User Experience and Acceptance, Impact on the Role of the Employee, Governance and Ethics, and Innovation and Organizational Transformation. Spearman’s rank-order correlations were estimated because the composite scores were derived from ordinal Likert-type indicators. Means and standard deviations were also calculated for each dimension.

## 4. Results

### 4.1. Validity Evidence Based on Test Content

The instrument’s content validity was assessed by an expert panel to ensure that the items adequately reflected the relationship between leadership and human–AI collaboration, as well as its conceptual and operational dimensions. This procedure allowed for the examination of the relevance, clarity, coherence, and representativeness of each item in relation to the proposed theoretical construct, considering that measuring this phenomenon requires capturing not only leadership styles but also practices linked to the strategy, ethics, sustainability, and governance of artificial intelligence in organizational contexts. Expert review is an essential phase in instrument development, as it helps ensure that the content is congruent with the conceptual definition and the intended empirical use of the instrument.

The panel consisted of nine experts with experience in leadership, organizational behavior, research methodologies, and studies on artificial intelligence applied to organizations. Each expert individually evaluated the items using a structured assessment matrix, considering criteria of relevance, wording, comprehensibility, and theoretical adequacy. Subsequently, the qualitative observations provided by the judges were analyzed to identify ambiguities, redundancies, formulation problems, and potential mismatches between the items and the model’s dimensions. This strategy is consistent with recommended procedures for content validation through expert judgment, especially in the construction of new scales or those adapted to emerging contexts.

Furthermore, the expert evaluation was aligned with the explanatory model of Zárate-Torres et al. (2025). Validation was not limited to verifying the wording of the items, but also their capacity to represent the eight dimensions related to the two constructs. This approach strengthens content validity by ensuring the correspondence between the instrument’s content and the underlying theoretical framework, thus facilitating its subsequent application in empirical studies on leadership in collaborative organizational environments mediated by AI (Zárate-Torres et al., 2025).

#### Descriptive Analysis

**Figure 1:**
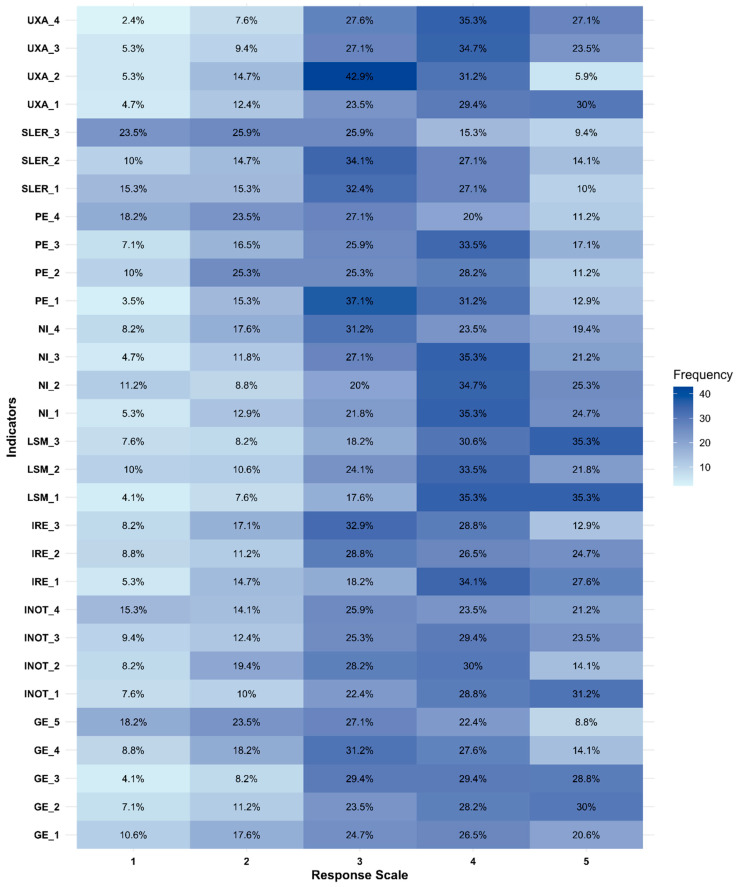
Distribution of responses.

The descriptive distribution of responses across the 30 items revealed a general tendency toward the middle and upper categories of the scale. Overall, response options 4 and 5 represented 49.6% of all observations, whereas category 3 accounted for 26.9%. By contrast, the lower categories (1 and 2) jointly represented 23.4% of the responses. Consistent with this pattern, the overall mean across items was 3.38, suggesting a moderately favorable assessment of the content captured by the instrument. At the item level, meaningful differences were observed in both central tendency and response distribution. The highest mean scores were recorded for LSM_1 (M = 3.90), LSM_3 (M = 3.78), and UXA_4 (M = 3.77), which also showed the largest proportions of responses in categories 4 and 5, reaching 70.6%, 65.9%, and 62.4%, respectively. In contrast, the lowest mean scores were found for SLER_3 (M = 2.61), GE_5 (M = 2.80), and PE_4 (M = 2.82), together with a greater concentration of responses in the lower categories. SLER_3 accumulated 49.4% of responses in categories 1 and 2, whereas GE_5 and PE_4 each reached 41.7%. These overall response patterns are presented in Figure 1.

**Figure 2:**
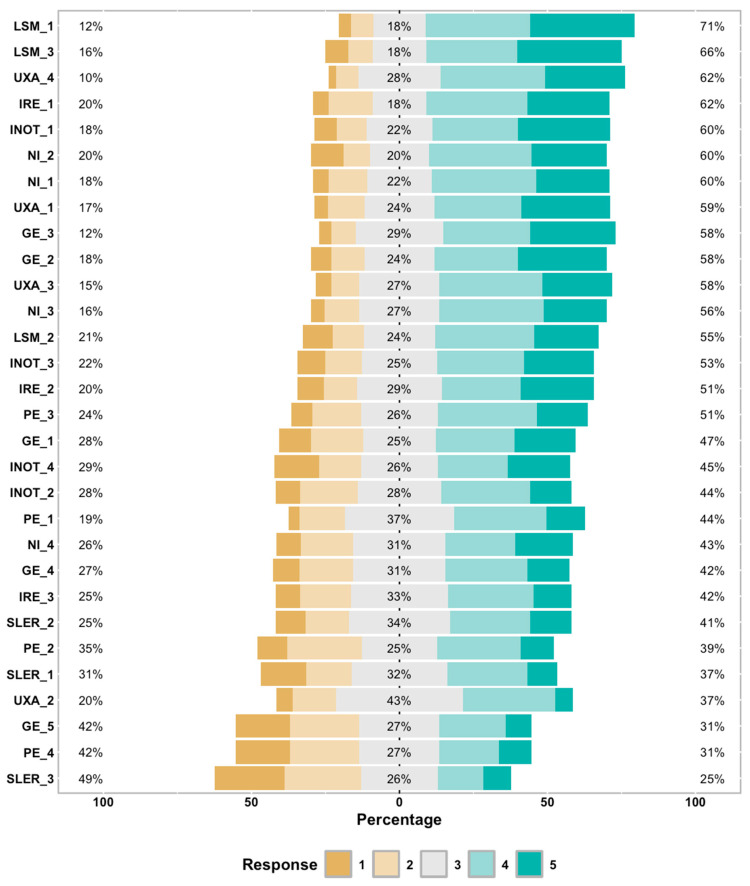
Concretion of responses.

Additional heterogeneity was observed in the concentration of responses in the midpoint category of the scale. This pattern was especially evident for UXA_2 (42.9% in category 3), followed by PE_1 (37.1%) and SLER_2 (34.1%), suggesting comparatively more neutral or less clearly defined perceptions regarding these items. Differences in response dispersion across items were also apparent. The highest standard deviations were observed for INOT_4 (SD = 1.34), GE_1 (SD = 1.27), NI_2 (SD = 1.27), PE_4 (SD = 1.26), and SLER_3 (SD = 1.26), indicating greater heterogeneity in the evaluation of these indicators. These results suggest that, although the instrument exhibited an overall tendency toward favorable responses, substantial differences remained across items. The detailed percentage distribution by item is shown in Figure 2.

### 4.2. Validity Evidence Based on the Internal Structure

#### 4.2.1. Exploratory Factor Analysis

Regarding the conditions for Leadership, the adequacy of the correlation matrix was assessed before conducting the exploratory factor analysis. The Kaiser–Meyer–Olkin index indicated good sampling adequacy (KMO = 0.82), with item-level MSA values ranging from 0.81 to 0.84. Bartlett’s test of sphericity was statistically significant, χ^2^(15) = 576.43, *p* < 0.001. These results supported the factorability of the data and justified proceeding with the exploratory factor analysis.

The leadership construct was theoretically composed of two first-order dimensions. The first EFA was estimated using a two-factor solution with ULS extraction and Oblimin rotation. The resulting structure clearly differentiated the two expected components, with each factor retaining three items. Items LSM_1, LSM_2, and LSM_3 loaded on the factor associated with Leadership and Strategic Management, whereas items SLER_1, SLER_2, and SLER_3 loaded on the factor associated with Sustainable Leadership and Environmental Responsibility. All retained items showed factor loadings above the established threshold of 0.40, with no problematic cross-loadings. Therefore, no items were removed, and no additional EFA estimations were required.

The EFA solution presented for the Leadership construct retained two factors and six items. This solution explained 66.8% of the total variance, with factor loadings ranging from 0.751 to 0.885. Communalities ranged from 0.527 to 0.827, indicating that the retained items shared an adequate proportion of variance with the extracted factors. The correlation between the two factors was 0.652, supporting the use of an oblique rotation and indicating that the two leadership-oriented dimensions were meaningfully related but empirically distinguishable.

Empirical factor-retention criteria supported the retained structure. The Kaiser–Guttman criterion indicated a two-factor solution, with eigenvalues of 3.735 and 1.001 for the first two factors. Parallel analysis also supported the retention of two factors. The two-factor solution was therefore retained because it was statistically supported, theoretically coherent, and consistent with the original conceptual distinction between LSM and SLER.

Regarding the conditions for Human–AI Collaboration, the adequacy of the correlation matrix was assessed before conducting the exploratory factor analysis. The Kaiser–Meyer–Olkin index indicated excellent sampling adequacy (KMO = 0.94), with item-level MSA values ranging from 0.89 to 0.97. Bartlett’s test of sphericity was statistically significant, χ^2^(276) = 2789.54, *p* < 0.001. These results supported the factorability of the data and justified proceeding with the exploratory factor analysis.

Because the Human–AI Collaboration construct was initially grounded in six theoretically defined first-order dimensions, the first EFA was estimated using a six-factor solution with ULS extraction and Oblimin rotation. This estimation showed that PE_3 and IRE_1 had problematic cross-loadings, whereas UXA_1, UXA_3, IRE_3, and GE_5 had factor loadings below the established threshold of 0.40. These six items were, therefore, removed.

The second EFA showed that PE_4 continued to present a loading below 0.40, leading to its exclusion. In the third EFA, UXA_2 also fell below the minimum loading criterion and was removed. The fourth EFA identified GE_4 as another item with a loading below 0.40 and an ambiguous pattern of association across factors, which justified its exclusion. The fifth EFA retained only items with acceptable loadings and no problematic cross-loadings. However, one of the extracted factors was defined by a single item, IRE_2, which compromised the stability and interpretability of the solution.

To address the instability of the six-factor exploratory structure, progressively more parsimonious solutions were examined. The sixth EFA was estimated as a five-factor solution, but UXA_4 and IRE_2 showed loadings below 0.40 and were removed. The seventh EFA showed that INOT_1 also failed to reach the minimum loading threshold and was excluded. Although the eighth EFA no longer presented problematic loadings in the five-factor structure, one factor was defined only by PE_2, indicating that the solution remained unstable. Therefore, the ninth EFA was estimated as a four-factor solution. In this model, INOT_2 showed problematic cross-loading and was removed. The tenth EFA retained only items with acceptable loadings, but one factor was defined exclusively by PE_1.

The eleventh EFA was estimated as a three-factor solution, in which PE_1 failed to load adequately on any factor and was therefore removed. The twelfth EFA, specified with three factors after removing PE_1, produced the most stable and interpretable exploratory solution, with no factor loadings below 0.40, no problematic cross-loadings, and no single-item factors.

The retained exploratory solution for the Human–AI Collaboration construct comprised three factors and ten items: NI_1, NI_2, NI_3, NI_4, PE_2, GE_1, GE_2, GE_3, INOT_3, and INOT_4. Sampling adequacy remained excellent in this reduced solution (KMO = 0.90), with item-level MSA values ranging from 0.83 to 0.95. Bartlett’s test of sphericity also confirmed the suitability of the reduced correlation matrix for factor analysis, χ^2^(45) = 1057.15, *p* < 0.001.

This exploratory solution explained 58.8% of the total variance. Factor loadings ranged from 0.444 to 0.928, and no retained item showed factor loadings below 0.40 or problematic cross-loadings. Communalities ranged from 0.358 to 0.833, indicating that most items shared an adequate proportion of variance with the extracted factors. Inter-factor correlations ranged from 0.542 to 0.707, supporting the use of an oblique rotation and indicating that the three exploratory factors were meaningfully related but empirically distinguishable.

Empirical factor-retention criteria suggested both more parsimonious and theoretically richer alternatives. Specifically, the Kaiser–Guttman criterion and the Scree plot indicated a two-factor solution, whereas parallel analysis supported a three-factor solution. The three-factor structure was therefore retained as the most interpretable exploratory representation of the observed covariance pattern.

However, the EFA was not treated as the sole criterion for determining the final measurement structure. The Human–AI Collaboration instrument was developed from an a priori theoretical model comprising six dimensions, and the reduced three-factor solution required the removal of fourteen indicators, substantially limiting the conceptual coverage of the construct. In addition, one of the exploratory factors was represented by only two indicators. Consequently, the three-factor solution was interpreted as an exploratory and parsimonious representation of the data rather than as the final validated measurement model. The original six-dimensional structure was subsequently evaluated through confirmatory factor analysis, considering model fit, theoretical coherence, content coverage, and interpretability.

#### 4.2.2. Confirmatory Factor Analysis

**Figure 3:**
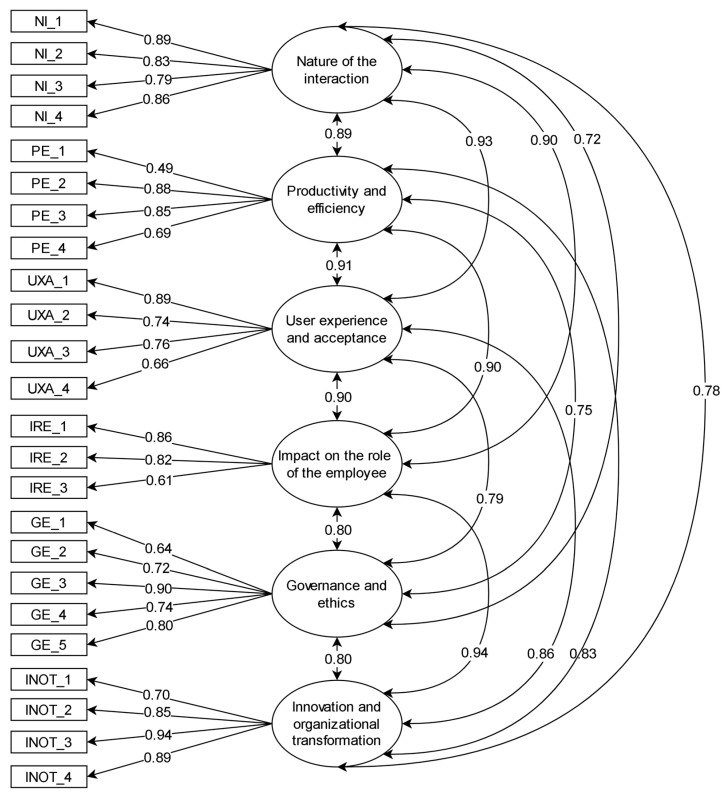
Standardized coefficients for Model 1 and their standard errors for Human–AI Collaboration. Note: Latent constructs are shown in ellipses, and observed variables are shown in rectangles. All coefficients are significant at *p* < 0.05. Source: Own elaboration.

For the Leadership construct, a CFA model (Table 1, Figure 3) was estimated based on the construct’s original theoretical specification. The model included two first-order factors: Leadership and Strategic Management [LSM_1–3] and Sustainable Leadership and Environmental Responsibility [SLER_1–3]. The model showed a favorable fit, with CFI = 0.994, TLI = 0.988, and SRMR = 0.035, whereas RMSEA was above the conventional threshold (0.090; 90% CI [0.038, 0.144]). Given the small degrees of freedom of the model, RMSEA was interpreted cautiously and evaluated together with the remaining fit indices. Nevertheless, the wide confidence interval, particularly its upper bound, indicates substantial uncertainty regarding the model’s approximate fit. Therefore, although the overall pattern of fit indices supports the two-factor structure, the RMSEA result should not be interpreted as providing unequivocal evidence of close fit.

**Figure 4:**
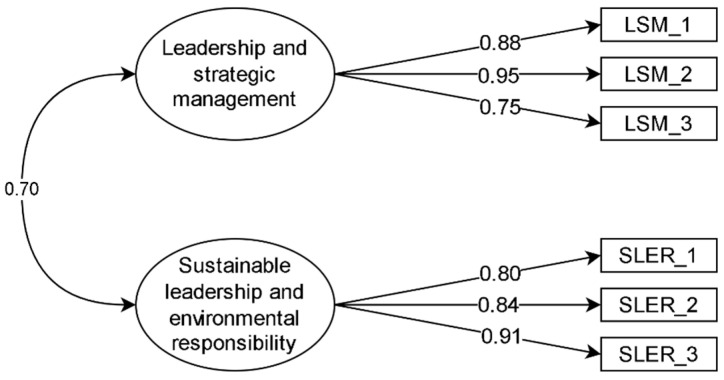
Standardized coefficients for Model 1 and their standard errors for Leadership. Note: Latent constructs are shown in ellipses, and observed variables are shown in rectangles. All coefficients are significant at *p* < 0.05. Source: Own elaboration.

Three CFA models were estimated and compared for the Human–AI Collaboration construct (Table 1). Model 1 (Figure 4) corresponded to the original theoretical specification of this construct and included six first-order factors: Nature of the Interaction [NI_1–4], Productivity and Efficiency [PE_1–4], User Experience and Acceptance [UXA_1–4], Impact on the Role of the Employee [IRE_1–3], Governance and Ethics [GE_1–5], and Innovation and Organizational Transformation [INOT_1–4]. Model 2 was derived from Model 1 after removing item PE_1, given its relatively low standardized loading. Model 3 was specified according to the EFA results and retained three factors, excluding items PE_1, PE_3, PE_4, UXA_1, UXA_2, UXA_3, UXA_4, IRE_1, IRE_2, IRE_3, GE_4, GE_5, INOT_1, and INOT_2.

Among the three models, Model 3 showed the strongest relative fit according to CFI = 0.988, TLI = 0.984, and SRMR = 0.040, whereas its RMSEA was slightly above the conventional threshold (0.083; 90% CI [0.056, 0.110]). The width of this interval, particularly its upper bound, indicates uncertainty regarding the model’s approximate fit. Model 1 also demonstrated favorable fit (RMSEA = 0.074, 90% CI [0.064, 0.084], SRMR = 0.057, CFI = 0.970, TLI = 0.965), as did Model 2 (RMSEA = 0.075, 90% CI [0.064, 0.086], SRMR = 0.055, CFI = 0.972, TLI = 0.967). The point estimates for Models 1 and 2 were within the acceptable range; however, their upper confidence limits slightly exceeded 0.08, suggesting that approximate fit should be interpreted with some caution.

Therefore, none of the three models provided unequivocal evidence of close fit based on RMSEA alone. Model 3 offered a more parsimonious representation and stronger relative fit on several indices, but it required the removal of fourteen indicators and substantially reduced the conceptual coverage of the construct. By contrast, Model 1 met the established fit criteria overall and preserved the complete six-dimensional structure proposed in the theoretical model. Model 2 did not produce a substantively meaningful improvement over Model 1 after removing PE_1. Therefore, Model 1 was retained as the final measurement model for Human–AI Collaboration, whereas Models 2 and 3 were interpreted as comparative and sensitivity analyses.

These results indicate that the exploratory and confirmatory findings are not mutually exclusive. The EFA revealed substantial empirical overlap among several dimensions, whereas the CFA showed that the six theoretically defined factors remained statistically tenable as distinct first-order components of Human–AI Collaboration.

Table 2 presents the standardized factor loadings, standard errors, z-values, significance levels, and explained variance (R^2^) for the CFA measurement models corresponding to Model 1 of the Leadership construct and Model 1 of the Human–AI Collaboration construct. In both models, all freely estimated factor loadings were positive and statistically significant (*p* < 0.001), supporting the association between the indicators and their respective latent constructs.

For the leadership construct, standardized loadings ranged from 0.753 to 0.947, indicating that the indicators were strongly associated with their respective latent constructs. The highest loading was observed for SLER_2 on Sustainable Leadership and Environmental Responsibility (SLER; λ = 0.947), whereas the lowest loading was observed for SLER_3 on the same factor (λ = 0.753). The R^2^ values ranged from 0.568 to 0.898, indicating that the model explained a substantial proportion of variance in the observed indicators.

The latent correlation between Leadership and Strategic Management and Sustainable Leadership and Environmental Responsibility was 0.702, suggesting that both dimensions were strongly related but empirically distinguishable.

For the Human–AI Collaboration construct, standardized loadings ranged from 0.491 to 0.941. The highest loadings were observed for INOT_3 on Innovation and Organizational Transformation (INOT; λ = 0.941), GE_3 on Governance and Ethics (GE; λ = 0.895), and NI_1 on Nature of the Interaction (NI; λ = 0.893). The lowest loading was found for PE_1 on Productivity and Efficiency (PE; λ = 0.491). Although PE_1 contributed less strongly to its latent factor than the remaining indicators, its loading was positive, statistically significant, and close to the conventional 0.50 reference value. Removing PE_1 in Model 2 produced only marginal changes in the global fit indices and did not improve RMSEA. The R^2^ values ranged from 0.241 to 0.885, indicating variability in the extent to which the indicators were explained by their underlying factors.

These results support the adequacy of the proposed first-order measurement models for both constructs, while also revealing differences in the relative contribution of individual items across dimensions.

#### 4.2.3. Discriminant Validity

Discriminant validity was assessed using the Fornell–Larcker criterion and the heterotrait–monotrait ratio of correlations (HTMT). The results are presented in Table 3. For the Leadership construct, discriminant validity was supported. The latent correlation between Leadership and Strategic Management and Sustainable Leadership and Environmental Responsibility was 0.702, which was lower than the square roots of their respective AVE values (0.850 and 0.863). The corresponding HTMT value was 0.678, remaining below the strict threshold of 0.85. For the Human–AI Collaboration construct, the evidence was mixed.

The Fornell–Larcker criterion was satisfied for only a limited number of factor pairs, indicating substantial shared variance among several dimensions. The HTMT results provided comparatively stronger support: seven factor pairs met the strict threshold of 0.85, and six additional pairs remained below the more flexible threshold of 0.90. However, the pairs Nature of the Interaction–User Experience and Acceptance (HTMT = 0.902) and Impact on the Role of the Employee–Innovation and Organizational Transformation (HTMT = 0.964) exceeded the recommended threshold. These findings indicate that discriminant validity was supported for several dimensions but remained limited for some highly associated factor pairs.

### 4.3. Reliability

The internal consistency of the dimensions associated with Leadership and Human–AI Collaboration was evaluated using Cronbach’s alpha, ordinal alpha, omega, omega2, and omega3. The results showed satisfactory reliability across all dimensions of the instrument (Table 4).

For the leadership construct, the reliability coefficients also showed adequate internal consistency. Leadership and Strategic Management (LSM) presented Cronbach’s alpha = 0.86, ordinal alpha = 0.89, omega = 0.85, omega2 = 0.85, and omega3 = 0.85. Sustainable Leadership and Environmental Responsibility (SLER) presented Cronbach’s alpha = 0.86, ordinal alpha = 0.90, omega = 0.87, omega2 = 0.87, and omega3 = 0.88. These results suggest that both leadership-oriented dimensions had stable and satisfactory internal consistency. Because all reliability coefficients exceeded the minimum recommended threshold of 0.70 (Nunnally & Bernstein, 1994), the reliability of the measurement dimensions can be considered satisfactory.

For the dimensions belonging to the human–AI collaboration construct, Cronbach’s alpha values ranged from 0.763 to 0.881, whereas ordinal alpha values ranged from 0.792 to 0.906. Omega and omega2 coefficients ranged from 0.784 to 0.891, while omega3 ranged from 0.778 to 0.921. These values indicate acceptable/good reliability across the dimensions associated with Nature of the Interaction (NI), Productivity and Efficiency (PE), User Experience and Acceptance (UXA), Impact on the Role of the Employee (IRE), Governance and Ethics (GE), and Innovation and Organizational Transformation (INOT).

Following commonly used benchmarks for internal consistency, the results indicate acceptable/good reliability, with some dimensions reaching very good levels, as shown in Table 4. In addition, the average variance extracted (AVE) ranged from 0.55 to 0.75 across the dimensions. Since all AVE values exceeded the recommended threshold of 0.50, the results also provide evidence of adequate convergent validity for the retained constructs.

### 4.4. Bivariate Correlations Among the Study Dimensions

Table 5 shows the descriptive statistics and Spearman bivariate correlations among the eight dimensions included in the instrument. This analysis was conducted to examine the associations among the dimensions derived from the proposed explanatory model. All correlations were positive and statistically significant at *p* < 0.001, with coefficients ranging from 0.500 to 0.783. The two Leadership dimensions were moderately correlated with each other (ρ = 0.510) and showed positive associations with the six Human–AI Collaboration dimensions.

Leadership and Strategic Management showed its strongest associations with Innovation and Organizational Transformation (ρ = 0.722) and Governance and Ethics (ρ = 0.678). Sustainable Leadership and Environmental Responsibility showed its strongest association with Productivity and Efficiency (ρ = 0.606). Within Human–AI Collaboration, the strongest correlations were observed between Impact on the Role of the Employee and Innovation and Organizational Transformation (ρ = 0.783), Nature of the Interaction and User Experience and Acceptance (ρ = 0.762), and User Experience and Acceptance and Innovation and Organizational Transformation (ρ = 0.728).

These results indicate that the dimensions were meaningfully interconnected and that the observed associations were consistent with the theoretical expectation that Leadership and Human–AI Collaboration are related organizational domains. These findings provide preliminary predictive validity evidence based on relationships among theoretically related dimensions within the instrument.

## 5. Conclusions

The study carried out allows us to confirm that the latent variables Leadership and Strategic Management (LSM) and Sustainable Leadership and Environmental Responsibility (SLER) measure Leadership. Likewise, the six-factor solution for Human–AI Collaboration, comprising Nature of the Interaction (NI), Productivity and Efficiency (PE), User Experience and Acceptance (UXA), Impact on the Role of the Employee (IRE), Governance and Ethics (GE), and Innovation and Organizational Transformation (INOT), measure the Human-AI Collaboration. Therefore, the proposed measurement scale allows us to contrast the approach of the latent variables for each studied construct, exposed by Zárate-Torres et al. (2025) in their explanatory model, managing to determine, with the application of the CFA, the items that evaluate each latent variable that make up these constructs in Colombian companies.

This study makes a novel contribution by explicitly integrating two conceptual frameworks that have evolved in parallel—leadership and human-AI collaboration—overcoming the existing fragmentation between scales focused on AI use or trust and those focused on leadership styles. In this sense, the proposed scale does not conceive leadership as a general set of styles, but rather as practices related to the strategy, ethics, sustainability, and organizational management of AI in collaborative contexts.

The study complements and expands upon the explanatory model of Zárate-Torres et al. (2025), providing empirical evidence from the development and validation of the proposed measurement scale that establishes the relationship between leadership and human-AI collaboration, moving beyond descriptive or unidimensional approaches. Thus, it contributes to the literature by offering a validated tool that facilitates comparability and knowledge accumulation in AI-mediated organizational environments.

The items measure the constructs for which it was designed, making a significant contribution to the evaluation of Leadership and Human–AI Collaboration in Colombian companies. Likewise, scores on the scale of measurement show good to excellent reliability. The interpretations have validity evidence based on the content and internal structure of the scale. Therefore, this scale can be useful for measuring the variables studied in Colombian companies.

Considering the results obtained, it follows that the scale of measurement created for each construct (Leadership and Human-AI Collaboration) complies with the psychometric properties related to the reliability and validity required in the measuring instruments in social science and with what is reported in other studies (Guillén-Gámez et al., 2026; Perkins et al., 2024; Larasati et al., 2023; Seeber et al., 2020).

Therefore, the proposed scale is ideal for measuring the constructs analyzed in Colombian companies. This instrument can be used by researchers seeking to corroborate the Zárate-Torres et al. (2025) model in other realities than the Colombian one. In addition, it would help managers and business managers evaluate their firms’ internal processes, identify areas that need improvement, and reinforce practices that are considered appropriate.

Because of the contribution made by the proposed measurement scale, an upcoming investigation should be related to its application in other countries in organizations of different sizes and productive sectors, to corroborate its universality, because new empirical studies are needed to test the measurement scale and determine if the proposed latent variables can measure these constructs in organizations from other countries with different conditions to the country of study.

Considering that the psychometric properties of the measuring instruments are of the scores and not of the tests themselves, additional studies would be required in other sectors and types of organizations to know the operation of the measurement scale. Also, this study collected two sources of validity evidence. However, the validation process involves collecting a greater amount of evidence. In this sense, evidence based on the relationship with other variables, internal processes, or consequences is necessary to demonstrate the robustness of the instrument.

A further limitation concerns the discriminant validity of some Human–AI Collaboration dimensions, the use of the same sample for the exploratory and confirmatory analyses, the positive wording of all instrument items, and the absence of an independent external criterion to broadly assess predictive validity. Although the six-factor solution was retained because of its theoretical coherence, conceptual coverage, and adequate overall fit, the Fornell–Larcker and HTMT results indicated substantial overlap among several factor pairs. Nature of the Interaction and User Experience and Acceptance, as well as Impact on the Role of the Employee and Innovation and Organizational Transformation, showed limited discriminant validity. In addition, because the EFA and CFA were conducted using the same sample, the retained structure could not be independently cross-validated. The exclusive use of positively worded items may also have increased susceptibility to acquiescence or common response patterns. Although negatively worded items can introduce comprehension and method effects of their own, future studies should examine alternative or more neutrally worded formulations and assess potential response-style effects. Although the positive and statistically significant correlations among the study dimensions provide preliminary evidence based on relationships among theoretically related variables, they should not be interpreted as evidence of predictive validity in the strict sense because no external criterion, such as task performance or organizational performance, was included.

Finally, future research should apply structural equation models to examine the relationships between Leadership and Human–AI Collaboration and to determine the effects of these constructs on other organizational variables. Such studies would contribute to further evaluating the explanatory model proposed by Zárate-Torres et al. (2025).

## Figures and Tables

**Table 1:** Goodness-of-Fit indicators of the models for the measurement scale for all models.

Model	RMSEA [90% CI]	SRMR	CFI	TLI
*Leadership*				
Model 1	0.090 [0.038, 0.144]	0.035	0.994	0.988
*Human–AI Collaboration*				
Model 1	0.074 [0.064, 0.084]	0.057	0.970	0.965
Model 2	0.075 [0.064, 0.086]	0.055	0.972	0.967
Model 3	0.083 [0.056, 0.110]	0.040	0.988	0.984

**Table 2:** Factor loadings, significance levels, and explained variance for the final six-factor Human–AI Collaboration model and the two-factor Leadership model.

Variable	Unstandardized Loading	Standard Error	z-Value	*p*-Value	Standardized Loading	R^2^
*Leadership*						
LSM_1	1.000	0.000	NA	NA	0.805	0.647
LSM_2	1.038	0.060	17.186	<0.001	0.835	0.697
LSM_3	1.128	0.059	19.015	<0.001	0.907	0.823
SLER_1	1.000	0.000	NA	NA	0.878	0.771
SLER_2	1.079	0.037	28.882	<0.001	0.947	0.898
SLER_3	0.858	0.039	22.164	<0.001	0.753	0.568
*Human-AI collaboration*						
NI_1	1.000	0.000	NA	NA	0.893	0.797
NI_2	0.927	0.041	22.450	<0.001	0.827	0.685
NI_3	0.883	0.040	22.233	<0.001	0.789	0.622
NI_4	0.961	0.036	26.366	<0.001	0.858	0.736
PE_1	1.000	0.000	NA	NA	0.491	0.241
PE_2	1.792	0.236	7.591	<0.001	0.880	0.775
PE_3	1.731	0.234	7.415	<0.001	0.850	0.723
PE_4	1.407	0.199	7.072	<0.001	0.691	0.478
UXA_1	1.000	0.000	NA	NA	0.893	0.798
UXA_2	0.833	0.041	20.150	<0.001	0.744	0.554
UXA_3	0.855	0.044	19.459	<0.001	0.764	0.584
UXA_4	0.738	0.054	13.699	<0.001	0.659	0.435
IRE_1	1.000	0.000	NA	NA	0.859	0.738
IRE_2	0.953	0.041	23.280	<0.001	0.819	0.670
IRE_3	0.715	0.058	12.247	<0.001	0.614	0.377
GE_1	1.000	0.000	NA	NA	0.641	0.411
GE_2	1.126	0.088	12.836	<0.001	0.722	0.522
GE_3	1.396	0.122	11.489	<0.001	0.895	0.802
GE_4	1.150	0.103	11.179	<0.001	0.738	0.544
GE_5	1.245	0.119	10.423	<0.001	0.798	0.637
INOT_1	1.000	0.000	NA	NA	0.705	0.497
INOT_2	1.206	0.087	13.922	<0.001	0.850	0.722
INOT_3	1.335	0.088	15.096	<0.001	0.941	0.885
INOT_4	1.257	0.088	14.299	<0.001	0.886	0.785

**Table 3:** Discriminant validity.

Factor 1	Factor 2	Latent Correlation	√AVE Factor 1	√AVE Factor 2	Fornell–Larcker	HTMT	HTMT Interpretation
*Leadership*							
LSM	SLER	0.702	0.850	0.863	Supported	0.678	Meets strict criterion
*Human-AI collaboration*							
NI	PE	0.886	0.843	0.744	Not supported	0.873	Meets flexible criterion only
NI	UXA	0.930	0.843	0.770	Not supported	0.902	Not supported
NI	IRE	0.897	0.843	0.771	Not supported	0.853	Meets flexible criterion only
NI	GE	0.724	0.843	0.764	Supported	0.713	Meets strict criterion
NI	INOT	0.781	0.843	0.850	Supported	0.801	Meets strict criterion
PE	UXA	0.912	0.744	0.770	Not supported	0.890	Meets flexible criterion only
PE	IRE	0.887	0.744	0.771	Not supported	0.896	Meets flexible criterion only
PE	GE	0.746	0.744	0.764	Not supported	0.806	Meets strict criterion
PE	INOT	0.827	0.744	0.850	Not supported	0.863	Meets flexible criterion only
UXA	IRE	0.895	0.770	0.771	Not supported	0.841	Meets strict criterion
UXA	GE	0.793	0.770	0.764	Not supported	0.782	Meets strict criterion
UXA	INOT	0.862	0.770	0.850	Not supported	0.889	Meets flexible criterion only
IRE	GE	0.795	0.771	0.764	Not supported	0.785	Meets strict criterion
IRE	INOT	0.938	0.771	0.850	Not supported	0.964	Not supported
GE	INOT	0.804	0.764	0.850	Not supported	0.823	Meets strict criterion

**Table 4:** Reliability of measurements.

Variable	*n*-Items	Alpha	Ordinal Alpha	Omega	Omega2	Omega3	Average Variance Extracted (AVE)	Interpretation
*Leadership*								
LSM	3	0.86	0.89	0.85	0.85	0.85	0.72	Good
SLER	3	0.86	0.90	0.87	0.87	0.88	0.75	Good to excellent
*Human–AI Collaboration*								
NI	4	0.88	0.91	0.88	0.88	0.88	0.71	Good to excellent
PE	4	0.76	0.79	0.81	0.81	0.85	0.55	Acceptable to good
UXA	4	0.82	0.85	0.83	0.83	0.84	0.59	Good
IRE	3	0.77	0.81	0.78	0.78	0.78	0.60	Acceptable to good
GE	5	0.83	0.86	0.84	0.84	0.86	0.58	Good
INOT	4	0.87	0.90	0.89	0.89	0.92	0.72	Good to excellent

**Table 5:** Descriptive statistics and bivariate correlations among the study dimensions.

Dimension	Mean	SD	LSM	SLER	NI	PE	UXA	IRE	GE	INOT
LSM	3.71	1.04	—							
SLER	2.94	1.07	0.510 ***	—						
NI	3.50	1.01	0.537 ***	0.523 ***	—					
PE	3.15	0.88	0.545 ***	0.606 ***	0.709 ***	—				
UXA	3.56	0.85	0.591 ***	0.545 ***	0.762 ***	0.706 ***	—			
IRE	3.44	0.98	0.575 ***	0.500 ***	0.716 ***	0.694 ***	0.678 ***	—		
GE	3.32	0.93	0.678 ***	0.569 ***	0.564 ***	0.608 ***	0.640 ***	0.620 ***	—	
INOT	3.39	1.06	0.722 ***	0.564 ***	0.694 ***	0.726 ***	0.728 ***	0.783 ***	0.680 ***	—

Note: *** *p* < 0.001.

## Data Availability

The databases used in this study are available at https://github.com/rodzarate/IAM (accessed on 16 May 2026).

## Abbreviations

The following abbreviations are used in this manuscript:

AI	Artificial Intelligence
LSM	Leadership and Strategic Management
SLER	Sustainable Leadership and Environmental Responsibility
NI	Nature of the interaction
PE	Productivity and Efficiency
UXA	User Experience and Acceptance
IRE	Impact on the Role of the Employee
GE	Governance and Ethics
INOT	Innovation and Organizational Transformation

## Appendix A

**Table A1:** English and Spanish versions of the measurement scale on Leadership and Human-AI Collaboration.

*Code*	*Item*
	*Leadership*
LSM_1	Leaders make AI adoption decisions with a long-term strategic perspective. Los líderes toman decisiones sobre la adopción de IA con una perspectiva estratégica a largo plazo.
LSM_2	Leaders continuously update their skills in managing AI. Los líderes actualizan de forma continua sus competencias para gestionar la IA.
LSM_3	Leaders apply ethical principles when making AI-related decisions. Los líderes aplican principios éticos al tomar decisiones relacionadas con la IA.
SLER_1	Decisions regarding AI consider its long-term environmental and social impact. Las decisiones sobre IA consideran su impacto ambiental y social a largo plazo.
SLER_2	Sustainability values guide the adoption of AI technologies. Los valores de sostenibilidad orientan la adopción de tecnologías de IA.
SLER_3	AI is used to reduce energy consumption, emissions, and waste generation. La IA se utiliza para reducir el consumo energético, las emisiones y la generación de residuos.
	*Human-AI Collaboration*
NI_1	Employees collaborate with AI systems to improve decision-making and operational efficiency. Los empleados colaboran con sistemas de IA para mejorar la toma de decisiones y la eficiencia operativa.
NI_2	AI automates repetitive tasks and helps reduce errors. La IA automatiza tareas repetitivas y contribuye a reducir errores.
NI_3	AI is used to support creativity and innovation activities. La IA se utiliza para apoyar las actividades de creatividad e innovación.
NI_4	AI is regularly used to plan and improve processes. La IA se usa con regularidad para planear y mejorar los procesos.
PE_1	Digital technologies are routinely used to manage information in real time. Las tecnologías digitales se utilizan habitualmente para gestionar la información en tiempo real.
PE_2	AI is used to improve the availability of information for decision-making. La IA se utiliza para mejorar la disponibilidad de información para la toma de decisiones.
PE_3	AI is used to analyze the organizational environment and anticipate trends. La IA se utiliza para analizar el entorno organizacional y anticipar tendencias.
PE_4	The organization collaborates with academic or R&D institutions to strengthen AI capabilities. La organización colabora con instituciones académicas o de I + D para fortalecer las capacidades de IA.
UXA_1	I use AI to facilitate my daily work. Utilizo la IA para facilitar mi trabajo diario.
UXA_2	The reliability and accuracy of AI-generated results are verified before they are used. La confiabilidad y precisión de los resultados generados por la IA se verifican antes de ser utilizados.
UXA_3	A proper balance is maintained between human judgment and AI output. Se mantiene un equilibrio adecuado entre el juicio humano y los resultados de la IA.
UXA_4	Employees use AI as an opportunity to develop new professional skills. Los empleados utilizan la IA como una oportunidad para desarrollar nuevas habilidades profesionales.
IRE_1	AI is regularly integrated into work tasks. La IA se integra regularmente en las tareas laborales.
IRE_2	Training activities are conducted to develop AI-related technical and cognitive skills. Se realizan actividades de capacitación para desarrollar habilidades técnicas y cognitivas relacionadas con la IA.
IRE_3	Employees demonstrate the skills needed to adapt to AI-driven changes. Los empleados demuestran las habilidades necesarias para adaptarse a los cambios impulsados por la IA.
GE_1	Final responsibility for AI-supported decisions is assigned before AI is used. La responsabilidad final de las decisiones respaldadas por IA se asigna antes de que se utilice la IA.
GE_2	The use of AI adheres to ethical principles and institutional protocols. El uso de IA se ajusta a principios éticos y a los protocolos institucionales.
GE_3	AI is used in ways that preserve individual autonomy in decision-making. La IA se utiliza de forma que se preserva la autonomía individual en la toma de decisiones.
GE_4	The potential impacts of AI are assessed before implementation. Se evalúan los posibles impactos de la IA antes de su implementación.
GE_5	The trade-offs between algorithmic efficiency and human values are discussed collectively. Los dilemas entre eficiencia algorítmica y valores humanos se discuten de manera colectiva.
INOT_1	Senior management actively promotes AI experimentation and pilot projects. La alta dirección impulsa activamente la experimentación y los proyectos piloto con IA.
INOT_2	AI solutions are securely integrated across organizational functions. Las soluciones de IA están integradas de forma segura en todas las funciones organizativas.
INOT_3	AI is used to support collaborative work and improve organizational processes. La IA se utiliza para apoyar el trabajo colaborativo y mejorar los procesos organizativos.
INOT_4	AI is used to strengthen the organization’s competitiveness. La IA se utiliza para fortalecer la competitividad de la organización.
